# Preserving cognitive function for patients with overactive bladder: evidence for a differential effect with darifenacin

**DOI:** 10.1111/j.1742-1241.2008.01849.x

**Published:** 2008-11

**Authors:** G G Kay, U Ebinger

**Affiliations:** 1 Cognitive Research CorporationSaint Petersburg, FL, USA; 2 Novartis Pharmaceuticals CorporationEast Hanover, NJ, USA

## Abstract

**Background:**

Antimuscarinic agents used in the treatment of overactive bladder (OAB) differ in their potential to impair cognitive function. It is hypothesised that low brain concentrations and relatively low selectivity for the M_1_ muscarinic receptor may reduce the potential for adverse central nervous system (CNS) effects with darifenacin, compared with other antimuscarinics, particularly oxybutynin.

**Methods:**

Cognitive function studies evaluating darifenacin, oxybutynin, tolterodine, solifenacin and/or trospium were identified from publications databases (Medline, Biosis and Embase) and congress abstracts. Preclinical studies and randomised controlled trials in adults were reviewed.

**Results:**

Five randomised, double-blind, multiple-dose studies of cognitive function were identified. Oxybutynin was consistently associated with cognitive deficit (four studies), whereas darifenacin did not impair cognition (three studies). These findings were supported by data from sleep/attention and EEG studies. Tolterodine data were limited to one small study with each formulation. For solifenacin and trospium, there were no human studies evaluating memory, the cognitive function most vulnerable to CNS anticholinergics.

**Conclusions:**

There is compelling evidence of cognitive impairment with oxybutynin, whereas darifenacin stands out by demonstrating no impairment of memory or other cognitive functions in three randomised, controlled trials. This may be attributed to the differences in physicochemical properties, efflux mechanisms and relative M_1_ muscarinic receptor sparing. The risk of CNS impairment is of particular concern for vulnerable populations such as the elderly (a substantial proportion of the OAB population), and CNS-compromised neurogenic bladder patients such as those with multiple sclerosis or Parkinson’s disease.

**Review Criteria**

- Electronic publication databases (Medline, Biosis and Embase) were searched using the terms ‘muscarinic antagonists’ plus ‘mental processes/drug effects’ or ‘cognition’.
- Preclinical studies and clinical trials in adults were evaluated, focusing on the following agents: darifenacin, oxybutynin, solifenacin, tolterodine and trospium.
- Abstracts on these agents from key urology/incontinence congresses (International Consultation on Incontinence; International Continence Society; American Urological Association; European Association of Urology) between 2002 and 2007 were also examined.

**Message for the Clinic**

- Antimuscarinics used in the treatment of overactive bladder differ in their potential to affect cognitive function.
- In particular, treatment with agents that block M_1_ receptors in the brain are known to cause cognitive impairment.
- Darifenacin and tolterodine stand out as having been shown to not cause impairment of memory or other cognitive functions in randomised clinical trials.

## Introduction

Overactive bladder (OAB) is a common condition, comprising a symptom complex that includes urinary urgency, frequency and in one-third of patients, urgency-associated incontinence (1). The first-line pharmacological treatment for OAB is with antimuscarinics (2), which aim to reduce the frequency and intensity of involuntary contractions of the bladder detrusor muscle primarily via blockade of muscarinic M_3_ receptors (3,4). As with many medications, there is a delicate balance between the efficacy and the tolerability/safety profile of antimuscarinics. A thorough discussion of the physiological and pharmacological basis for the efficacy and the side effects of these medications was recently published (5). Attention has focused recently on the potential impact of these medications on the central nervous system (CNS). An important factor for consideration is whether the choice of antimuscarinic therapy can influence CNS outcomes, and evidence is accumulating for differences between antimuscarinic drugs in their potential to adversely affect memory and other aspects of cognitive function.

However, several other factors may also contribute to the CNS risk including patient age, concomitant conditions and associated treatments. These may affect CNS function individually or in a complex interplay when combined. The prevalence of OAB increases with advancing age (6,7), and therefore many patients receiving OAB antimuscarinic therapy are likely to have multiple comorbidities and to be taking several concomitant medications.

Thus, advancing age is itself associated with decline in cognitive function and increasing permeability of the blood–brain barrier, which can increase a patient’s susceptibility to the CNS effects of medications with anticholinergic effects, in the absence of other contributing factors (8). The presence of neurological conditions such as multiple sclerosis, Parkinson’s disease or stroke *per se* also increases the risk for developing cognitive dysfunction and is associated with increased permeability of the blood–brain barrier (9). As antimuscarinics are routinely prescribed for patients whose OAB symptoms arise from such neurological conditions, patients receiving antimuscarinic therapy may have comorbidities that may predispose them to further CNS effects from antimuscarinic therapy. In addition, medications given concomitantly for a variety of unrelated comorbid conditions may have ‘hidden’ anticholinergic effects, which add to the total anticholinergic burden on the patient and, consequently, impaired cognitive function. Indeed, multiple studies have reported a high prevalence of elevated anticholinergic load in elderly patients, and the cognitive consequences of this burden [e.g. (10–12)].

Because a substantial number of patients may be vulnerable to the adverse CNS effects of antimuscarinic therapy, efforts to minimise further risk are important. One factor for consideration is whether the choice of antimuscarinic therapy can influence CNS outcomes, because evidence is accumulating for the differences between antimuscarinic drugs in their potential to adversely affect cognition.

This paper discusses the pharmacological basis for these differences, and reviews the preclinical and clinical evidence for CNS effects of commonly used antimuscarinic agents (i.e. darifenacin, oxybutynin, tolterodine, solifenacin and trospium). These clinical data include studies of drug effects on cognition, sleep and EEG.

## Pharmacological basis for differential cognitive effects of antimuscarinic drugs

Antimuscarinic agents interact with receptors throughout the body, including the M_3_ receptors of the bladder detrusor muscle, which are thought to be the primary target for antimuscarinic therapy in OAB (3,4). In the CNS, all five of the known muscarinic receptor subtypes are expressed (5). Although the role of each subtype in the brain has not yet been fully elucidated, interactions with M_1_, M_2_ and M_4_ muscarinic receptor subtypes have each been implicated in cognitive impairment (5,8,13–15). However, the muscarinic M_1_ receptor subtype, in particular, is thought to play a crucial role in modulating cognitive function (5,8,13). Evidence for a dominant role for the M_1_ receptor includes the severe impairment of working memory seen in M_1_ knockout mice (mutants deficient in M_1_ receptors) and in animals administered intra-hippocampal injections of the M_1_ receptor antagonist pirenzepine (16–18), as well as the potential for muscarinic M_1_ agonist therapy to improve cognitive function in patients with dementia (19). By contrast, studies with M_3_ knockout mice have shown no impact on cognition or behaviour (20). Therefore, antimuscarinic therapy that is most selective for the M_3_ subtype combined with relative M_1_ sparing properties would be expected to have the lowest potential for adverse effects on cognition.

However, for an agent to exert a CNS effect, it must first reach the appropriate receptors in sufficient concentration. Thus, the extent to which an antimuscarinic agent can disrupt CNS function will depend upon several factors including (i) the ability of the drug to enter the brain, (ii) accumulation/retention within the brain in sufficient concentrations and (iii) interaction with muscarinic receptors within the brain, particularly M_1_ receptors.

### CNS penetration

Overactive bladder antimuscarinic agents differ with respect to their ability to penetrate the blood–brain barrier passively, and the extent to which they are actively transported across the blood–brain-barrier by transporter proteins such as P-glycoprotein and the multidrug-resistance-associated proteins (MRPs; e.g. MRP1–9). Passive penetration is the greatest for non-polar molecules of small molecular size and high lipophilicity (i.e. limited solubility in alcohol) (8,13). Oxybutynin, a relatively small (357 kDa), highly lipophilic molecule can readily cross the blood–brain barrier, whereas other antimuscarinics are considerably larger (e.g. tolterodine 475.6 kDa, solifenacin 480.6 kDa and darifenacin 507.5 kDa), a factor which would hinder CNS penetration (13). Similarly, trospium, a (428.0 kDa), hydrophilic quaternary ammonium antimuscarinic compound, can be expected to show low penetrative ability under normal conditions.

However, all antimuscarinic agents have the potential to cross the blood–brain barrier under certain circumstances, because the integrity of the barrier can become disrupted in the presence of a range of conditions such as diabetes, Alzheimer’s disease, stroke, trauma, multiple sclerosis, Parkinson’s Disease and advancing age (8,13,21–23). As reviewed elsewhere (8,13), the mechanisms involved in this increased permeability are diverse, and may include epithelial shrinkage and capillary dilatation in the brain. For example, trauma to the brain has been shown to induce the expression of junctional adhesion molecule, which appears to lead to the breakdown of blood–brain barrier (24). Under these conditions, the potential for drug penetration into the CNS is increased, and the ability of the drug to remain in the brain and interact with specific muscarinic receptors becomes critical.

### CNS accumulation

There are few preclinical publications evaluating the concentrations of antimuscarinic drugs in the brain. One study with oxybutynin evaluated CNS penetration in terms of muscarinic receptor occupancy in conscious rhesus monkeys, using positron emission tomography (25). After single oral (p.o.) doses of oxybutynin 0.1 or 0.3 mg/kg, peak plasma concentrations of oxybutynin and its active metabolite (*N*-desethyl-oxybutynin) were reached at 30 min (7.9 ± 7.1 and 29.1 ± 36.4 ng/ml at 0.1 mg/kg, and 21.9 ± 10.9 and 63.1 ± 53.6 ng/ml at 0.3 mg/kg respectively). At 1 h after the 0.1 and 0.3 mg/kg doses, muscarinic receptor occupancy in each brain region evaluated (frontal, temporal and occipital cortices, cingulate gyrus, caudate, amygdala, putamen, hippocampus, thalamus and cerebellum) was estimated to be about 40% and 60% respectively. These levels decreased at 4 h postdose, in line with plasma concentrations.

By contrast, two publications of tissue distribution in rodents administered ^14^C-labelled tolterodine or ^14^C-darifenacin indicated relatively low CNS penetration and accumulation of these agents (26,27). In mice administered ^14^C-tolterodine as single (4 or 12 mg/kg), or as repeated p.o. doses (12 mg/kg/day for 7 days), brain concentrations at 2 h postdose were 0.07, 0.45 and 0.99 μg equivalents/g, compared with plasma concentrations of 0.39, 1.08 and 1.47 μg equivalents/ml, respectively, i.e. brain concentrations ranged from 18% to 67% of those achieved in plasma at the same time points (26). Similarly, in rats given ^14^C-darifenacin 4 mg/kg intravenously (i.v.), brain concentrations were 66% and 29% of blood concentrations at 5 min and 1 h postdose (0.37 vs. 0.56 μg equivalents/g at 5 min, and 0.13 vs. 0.44 μg equivalents/g at 1 h) respectively (27). However, a second study in the same publication reported somewhat lower CNS penetration following p.o. administration of 10 mg/kg ^14^C-darifenacin in rats, with cervical spinal fluid concentrations in pooled samples obtained at 1 and 4 h postdose approximately 10% of those in plasma dialysate (14.5 vs. 139.3 μg equivalents/ml).

As drug accumulation in the brain is dependent not only on passive penetration through the blood–brain barrier, but also on persistence within the brain, it is of interest that there are specific efflux mechanisms. Darifenacin transport is mediated by P-glycoprotein (28), and that of trospium chloride by one of the MRPs (29), while there are no known active mechanisms for other OAB antimuscarinics. These active transport mechanisms reduce the potential for the drug to accumulate and remain within the CNS and may contribute to the observation of very low penetration of ^14^C-darifenacin into the brain relative to other tissues (27).

While CNS concentrations of an antimuscarinic agent are important, an additional consideration is the additive CNS impact of an elevated drug burden associated with the use of multiple medications with anticholinergic activity (11). This is a particular concern for the older patient, as this is the population most subject to polypharmacy, often including the use of inappropriate medications with anticholinergic effects (30). In older patients, particular care is warranted to limit the anticholinergic load that may contribute to cognitive impairment.

### CNS muscarinic receptor binding

Following drug penetration and accumulation within the brain, the ability of an agent to block critical M_1_ receptor sites is a key factor contributing to drug-related cognitive dysfunction. Several studies have compared the OAB antimuscarinics to determine their relative *in vitro* binding selectivity for different muscarinic receptor subtypes (31–40). These studies have shown that darifenacin demonstrates consistently high relative selectivity for the M_3_ receptor subtype (which is presumed to be the primary target for OAB therapy) over the other receptor subtypes. Darifenacin demonstrated a selectivity ratio of 9.3 : 1 for the M_3_ receptor over the M_1_ subtype in a comparative study by Napier and Gupta (33), and 16 : 1 in a more recent competitive binding study (35). By contrast, other antimuscarinic agents were consistently found to be relatively non-selective for M_3_ receptors, with ratios of binding for M_3_ over M_1_ receptors ranging from 0.5 (i.e. a twofold greater binding affinity for M_1_ than M_3_ receptors) to 2.5 across all studies (31–40).

Overall, these findings suggest that the potential for negative cognitive effects among all currently available OAB drugs is likely to be the highest for oxybutynin, which demonstrates a high propensity for CNS penetration and accumulation coupled with non-selectivity for M_3_ receptors over the M_1_ receptor subtype. By contrast, for darifenacin, a drug with low CNS perfusion (arising from limited penetration and active efflux from the brain) and which has relatively low affinity for muscarinic M_1_ receptors, the potential for adverse CNS effects would be expected to be much lower. In order to confirm the clinical relevance of these differences in penetration, accumulation and selectivity profiles, data from specific cognitive function studies and sleep/EEG studies with OAB antimuscarinics are reviewed below, focusing in particular on studies with oxybutynin and darifenacin.

## Cognitive effects of OAB antimuscarinics in animal studies

Several papers have reported the behavioural effects in rodents of antimuscarinic drugs currently used for the treatment of OAB (41–44). Three studies evaluated the effects of oxybutynin in rats, using propiverine as a comparator and/or scopolamine as a positive control, and in each study, oxybutynin administration was associated with significant impairment of memory. In the most recent study, tolterodine had no effect on memory in mice undergoing a passive-avoidance test at 1 or 3 mg/kg p.o. (doses resulting in concentrations estimated be up to six times the therapeutic levels in man), in contrast to the memory impairment (decreased latency) observed with scopolamine 3 mg/kg (44). Darifenacin was evaluated only in one recent study and, in contrast to oxybutynin, was not associated with cognitive deficits (43). In this study, antimuscarinic agents (oxybutynin, darifenacin, tolterodine, solifenacin, propiverine or scopolamine) were administered i.v. to rats 10 min before an initial passive-avoidance task (acquisition) and latency time was measured when the task was repeated 24 h later (retention). Administration of oxybutynin (0.1–1 mg/kg), propiverine (1–10 mg/kg) or scopolamine (0.1–1 mg/kg) significantly impaired memory retention, seen as dose-dependent reductions in latency. Tolterodine had no effect at lower doses (0.3 or 0.1 mg/kg), but showed a trend for impaired learning at the highest dose (1 mg/kg; p = 0.054 for the reduction in latency), although this represents 100× the doses of tolterodine required to affect bladder contractions (assessed as inhibition of carbachol-induced increase in intravesical pressure). By contrast, darifenacin (0.1–1 mg/kg) and solifenacin (0.3–3 mg/kg) did not affect retention even at the highest doses, which represented 102× and 130× the doses required to inhibit carbachol-induced bladder contraction respectively.

The extent to which these animal models translate into clinical differences in CNS effects between commonly used OAB antimuscarinics is examined further by a review of published data from controlled clinical trials.

## Cognitive effects of OAB antimuscarinics in double-blind clinical studies

Several prospective, randomised, double-blind, clinical studies have evaluated the effects of antimuscarinic drugs on cognitive function at steady state (i.e. after at least 7 days of dosing), as summarised in Table 1 (45–49). Oxybutynin was evaluated in four studies involving a total of 315 subjects, and was consistently associated with deterioration in cognitive function. By contrast, darifenacin administration compared with placebo, in three studies involving a total of 302 subjects, resulted in no significant effect on learning or memory in healthy adults (45–47).

**Table 1:** Overview of cognitive function studies evaluating OAB antimuscarinic agents in adults

References	Study design, tests and patients	Treatments	Key outcomes
**Multiple-dose studies**			
Kay et al. (47)	Randomised, double-blind, parallel group, multicentre study (3 weeks of treatment) Computerised CFT (10 tests) performed at baseline and weeks 1, 2 and 3 150 healthy men and women (60–83 years)	Darifenacin (*n*=49): 7.5 mg/day (weeks 1 and 2) then 15 mg/day (week 3) Oxybutynin ER (*n*=50): 10 mg/day (week 1), 15 mg/day (week 2), 20 mg/day (week 3) Placebo (*n*=51): weeks 1–3	Delayed recall (NFAT) at week 3 not significantly different between darifenacin and placebo Delayed recall (NFAT) at week 3 significantly impaired with oxybutynin (p < 0.05 vs. placebo or vs. darifenacin) comparable to 16 years of brain ageing No between-group differences in self-rated memory (i.e. subjects unaware of memory deterioration)
Kay and Wesnes (45)	Randomised, double-blind, 4-way cross-over study (7-day treatment and 7-day washout periods) Computerised CFT (12 variables) and EEG recordings performed at baseline and day 7 of each treatment 23 healthy men (19–44 years)	Darifenacin 7.5 mg/day Darifenacin 15 mg/day Dicyclomine (positive control: M_1_ selective antimuscarinic) 20 mg qid Placebo	No significant effect on CFT with either dose of darifenacin and no clinically relevant effects on EEG Impaired performance on 5/12 variables at 2 h postdose with dicyclomine accompanied by EEG slowing
Lipton et al. (46)	Randomised, double-blind, 3-period crossover study (14-day treatment and 7-day washout periods), each subject receiving 3 of 5 treatments Computerised CFT (5 tests) at baseline and week 2 of each treatment period 129 healthy men and women (65–84 years)	Darifenacin 3.75 mg/day (*n*=65) Darifenacin 7.5 mg/day (*n*=70) Darifenacin 15 mg/day (*n*=61) Darifenacin IR* 5 mg tid (*n*=65) Placebo (*n*=66)	Darifenacin not significantly different from placebo for primary end-points of CFT (MSS, SCRT, WRS) at any dose No changes in self-rated alertness or contentment with darifenacin vs. placebo
Kay et al. (47)	Randomised, double-blind, crossover study (2 × 3-week treatment periods with 7 days of washout) Computerised CFT performed at baseline and 3 weeks 22 healthy men and women (mean age 63 years)	Tolterodine ER 4 mg/day (weeks 1–3, with sham titration) Oxybutynin ER: 10 mg/day (week 1), 15 mg/day (week 2), 20 mg/day (week 3)	No significant change in delayed recall (NFAT) or other outcome measures from baseline to week 3 of tolterodine treatment Delayed recall (NFAT) at week 3 significantly impaired with oxybutynin vs. baseline comparable to 20 years of ageing Delayed recall performance significantly worse with oxybutynin ER than tolterodine ER at week 3 but not at earlier time points No awareness of changes in memory at any time point
Nagels et al. (49)	Randomised, double-blind, crossover study (2 × 8-week treatment periods) CFT included PASAT and ADAS-Cog tests; MACFIMS and MMSE were also assessed 14 patients with MS (ages not specified)	Oxybutynin IR 2.5 mg tid Tolterodine IR 2 mg bid	Tolterodine was associated with a trend to better performance on PASAT than oxybutynin ADAS-Cog and MMSE did not differ between treatment periods
**Single-dose studies**			
Katz et al. (50)	Randomised, double-blind, placebo-controlled cross-over study (single doses with 1-week washout) Combination of pencil and paper, interview and computerised CFT (15 tests lasting 1 h), starting 90 min postdose 12 healthy men and women (75–76 years)	Oxybutynin HCl† 5 mg Oxybutynin HCl† 10 mg Diphenhydramine HCl† (positive control: antihistamine with known anticholinergic and cognitive effects) 50 mg Placebo†	Oxybutynin at both doses caused significant decrements on 7/15 cognitive measures Diphenhydramine caused significant decrements on 5/15 cognitive measures Effects of oxybutynin remained significant after Bonferroni correction
Diefenbach et al. (52)	Randomised, double-blind, placebo-controlled, cross-over study (single doses with 8-day washout) Sleep study with additional assessment of reaction time (ZVT) and attention (d2 test) 1 h postdose 24 healthy men and women (51–65 years)	Trospium 45 mg Oxybutynin IR 15 mg Tolterodine IR 4 mg Placebo	No significant differences between any drug and placebo in reaction time on ZVT, or number of items completed/mistakes or target items missed in d2 test
Diefenbach et al. (51)	Randomised, double-blind, placebo-controlled, cross-over study (single doses with 8-day washout) Sleep study with additional assessment of reaction time (ZVT) and attention (d2 test) 1 h postdose 24 healthy men and women (22–36 years)	Trospium 45 mg Oxybutynin IR 15 mg Tolterodine IR 4 mg Placebo	No significant differences between any drug and placebo in reaction time on ZVT, or number of items completed/mistakes or target items missed in d2 test

* Non-marketed formulation. †Administered as liquids diluted to 100 ml in fruit juice. ADAS-Cog, Alzheimer’s disease assessment scale, cognitive subscale; bid, twice daily; CFT, cognitive function tests; ER, extended release; HCl, hydrochloride salt; IR, immediate release; MACFIMS, Minimal Assessment of Cognitive Function in Multiple Sclerosis; MMSE, Mini-Mental State Examination; MS, multiple sclerosis; MSS, memory scanning sensitivity; NFAT, name–face association test; PASAT, paced auditory serial addition test; qid, four times daily; SCRT, speed of choice reaction time; tid, three times daily; WRS, word recognition sensitivity; ZVT, Zahlen–Verbindungs test.

All three darifenacin studies assessed cognition using a battery of computerised tests, and evaluated both doses approved for the treatment of OAB (7.5/15 mg once daily). Two of the studies involved a large number of older subjects (*n*=129 and 150, ≥ 60 years of age). In a study conducted with younger adults (*n*=27, age 19–44 years), each treatment (darifenacin 7.5 and 15 mg, dicyclomine, and placebo) was administered for 1 week (45). The two clinical doses of darifenacin were compared with the M_1_-selective muscarinic antagonist dicyclomine (used as a positive control). Although darifenacin had no effect on memory or other cognitive functions, dicyclomine resulted in significant impairment of cognitive function, which was observed on five of the 12 cognitive function variables assessed (45). In the second study, older subjects (*n*=129, age 65–84 years) received either darifenacin (7.5 mg and 15 mg/day) or placebo (46). Test results showed that performance on cognitive testing was comparable for the two clinical doses of darifenacin and placebo.

In a third study, the effects of darifenacin on cognitive function were compared with those of both oxybutynin extended release (ER) and placebo (*n*=150, age 60–83 years) (47). Dose escalation was conducted according to the US prescribing information of both drugs.

At both doses (7.5 and 15 mg), darifenacin had no significant effects on memory compared with placebo. By contrast, oxybutynin ER resulted in deterioration in memory over time, with significant differences observed from week 1 (at 10 mg/day) for secondary measures and from week 2 (at 15 mg/day) for the primary outcome measure (delayed recall on the Name–Face Association Test) (47). In spite of the significant decline in memory performance, which is equivalent to that of 16 years of cognitive ageing, participants receiving oxybutynin ER were not aware of any change in their memory.

Cognitive function studies with other OAB agents in adults are limited, and each involves relatively small numbers of subjects (Table 1). Two small studies, which are available only as published abstracts, have compared the CNS effects of tolterodine and oxybutynin at steady state. The more recent of these was a double-blind, 3-week, cross-over study (*n*=22) in older adults (mean age 63 years) comparing tolterodine ER (4 mg/day) with oxybutynin ER (10–20 mg/day). The study showed that oxybutynin ER 20 mg/day impaired cognitive performance relative to baseline, whereas no decline was seen with tolterodine ER (48). The other study compared the cognitive effects of oxybutynin immediate release (IR) (2.5 mg three times daily) with tolterodine IR (2 mg twice daily) in 14 patients with multiple sclerosis who also had complaints of OAB and cognitive difficulties (49). The results of this cross-over study indicated a trend towards better performance during treatment with tolterodine compared with oxybutynin.

Three further studies evaluated the effects of single doses of IR oxybutynin in small numbers of subjects (Table 1) (50–52). The first of these compared the acute effects of IR oxybutynin (5 or 10 mg), diphenhydramine (50 mg) and placebo on cognitive function in older adults (≥ 65 years) (50). The effect of oxybutynin appeared to be at least as great as that of diphenhydramine, an antihistamine with marked anticholinergic activity that served as the positive control for the study. The remaining two studies were primarily evaluations of sleep effects (see below) in healthy young (*n*=24, 22–36 years) and older volunteers (*n*=24, 51–65 years) but also assessed some cognitive parameters (51,52). Both studies compared the effects of single doses of trospium (45 mg) and oxybutynin IR (15 mg) or tolterodine IR (4 mg). Reaction time, assessed using the Zahlen–Verbindungs Test (a number combination test) and attention/concentration (the d2 test) evaluated at 1 h postdose showed no significant differences between these treatments and placebo. However, the clinical relevance of these findings is limited by the study design, in which cognitive effects were evaluated at a single time point following administration of a single dose and did not include an assessment of memory.

## Effects of OAB antimuscarinics in sleep and EEG studies

Several studies have evaluated the effects of OAB antimuscarinics on brain biomarkers including sleep and/or EEG. Although the link between such effects and cognitive function is unclear, these studies can be used as indicators of the potential effect of a drug on brain function. Overall, the results support the study findings reviewed above suggesting differential effects of OAB antimuscarinics on cognitive function.

### Sleep parameters

Two randomised, double-blind, cross-over studies compared the effects of single doses of oxybutynin IR (15 mg), tolterodine IR (4 mg), trospium (45 mg) and placebo on sleep in healthy young (22–36 years) or older (51–65 years) subjects (51,52). In both studies, oxybutynin resulted in reductions in rapid eye movement (REM) sleep as a proportion of total sleep time (p < 0.05 vs. trospium in younger subjects; p = 0.002 vs. placebo in older subjects), accompanied by increases in REM latency (p = 0.03 vs. placebo, p = 0.001 vs. trospium, p = 0.045 vs. tolterodine in younger subjects; not significant in older subjects). The proportion of REM sleep was also reduced by tolterodine vs. placebo in older subjects (p = 0.0002), but was not significantly altered in younger subjects. Further evaluation in a pooled analysis of these two studies indicated that tolterodine also reduced the proportion of REM sleep in subjects who were classified as poor metabolisers or intermediate metabolisers based on CYP2D6 status (53). No studies have been published evaluating sleep parameters during darifenacin administration.

### Quantitative EEG studies

Two quantitative EEG studies in healthy volunteers compared the effects of oxybutynin and trospium, the more recent of which also evaluated tolterodine (54,55). In the earlier open-label study, 12 healthy men (26 ± 4 years) received single doses of oxybutynin (20 mg) or trospium chloride either i.v. (1.2 mg) or orally (45 mg), at intervals of at least 6 days. Oxybutynin significantly altered quantitative EEG parameters during eyes-open, eyes-closed and reaction-time test periods, whereas i.v. trospium led to a marginal decrease only during the eyes-closed period, and oral trospium resulted in no significant changes (54). In the more recent study, 64 healthy men (18–35 years) received trospium (15 mg × 3 doses), oxybutynin (5 mg × 3 doses) or tolterodine (2 mg × 2 doses) each for 1 day (doses given at 5-h intervals), in a single-blind, cross-over design (55), and EEG was recorded at baseline and up to 4 h after each dose. Trospium or tolterodine administration did not produce any important changes in quantitative EEG parameters compared with placebo, whereas oxybutynin caused significant power reductions in four frequency bands.

The EEG effects of darifenacin have also been reported (without quantitative analysis) in one of the cognitive function studies discussed earlier (45). Placebo or darifenacin administration (7.5 or 15 mg/day for 7 days) resulted in no clinically relevant effect on EEG, whereas the positive control (dicyclomine) resulted in EEG slowing.

## Summary and conclusions

The risk of cognitive impairment during antimuscarinic therapy for OAB is an important concern, particularly for those with comorbid conditions that may impair CNS function and which are frequently associated with concomitant neurogenic bladder. Also included are persons taking multiple medications, with anticholinergic activity, which contribute to the anticholinergic load. It is noteworthy, that drug-induced deterioration in memory and the effects on other cognitive processes are often unnoticed and unreported by the patient.

Evaluation of the pharmacological mechanisms that could contribute to drug-induced effects on cognition suggests that antimuscarinic OAB agents differ in their ability to penetrate the blood–brain barrier, accumulate and interact with the M_1_ receptor in the brain. Amongst the antimuscarinic agents evaluated, darifenacin displays the greatest relative selectivity for the M_3_ receptor over the M_1_ subtype, whereas all other agents were relatively non-selective.

In this review, five randomised, controlled trials investigating cognitive function under OAB antimuscarinics administration in steady-state conditions have been reviewed (45–49). Substantial differences exist between the individual agents. In particular, oxybutynin has consistently been shown to cause deterioration in memory, the parameter considered to be the most sensitive to anticholinergic effects. In contrast, no such impairment was seen with darifenacin in the three studies which investigated this agent. Similarly, no cognitive decline was seen with tolterodine in two multiple-dose studies (48,49), although the findings need to be interpreted with caution because of the small number of subjects and the absence of a placebo control group. In addition, these two studies used different formulations of tolterodine (i.e. immediate and ER).

Impairment of CNS functioning was also seen in studies evaluating the effects of single doses of oxybutynin on cognition (50–52), sleep, and EEG (51–56), whereas trospium, was found to be free of impairment of attention, sleep and EEG parameters. Unfortunately, there are no known studies investigating the effect of trospium on memory.

Overall, this review indicates that a considerable body of preclinical and clinical data has accumulated to suggest that oxybutynin can cause cognitive impairment, which is further supported by the findings from sleep studies and quantitative EEG analyses. Indeed, the strength of evidence of an increased risk of CNS adverse events with oxybutynin has been recognised by the US Food and Drug Administration, resulting in new precautions in the labels for oxybutynin-containing products. Under the heading, Central Nervous System Effects, the new labels state that ‘patients should be monitored for signs of anticholinergic CNS effects, particularly in the first few months after beginning treatment or increasing the dose’ (57).

Conversely, amongst the OAB antimuscarinics evaluated, darifenacin was found to have the largest body of evidence demonstrating no impairment of memory or other cognitive functions in younger and older adults. This evidence is fully consistent with the darifenacin profile, which is characterised by its low CNS penetration and accumulation, in addition to its relative M_1_ receptor sparing properties.

In conclusion, the findings from this review indicate that appropriate selection of OAB medications is important in order to minimise the risk of CNS effects and enable long-term treatment for OAB with confidence in the safety of the therapy. Further long-term evaluation and postmarketing studies are awaited to confirm this safety profile in clinical practice.
